# Acupoint catgut embedding for asthma in adults: Protocol for a systematic review

**DOI:** 10.1097/MD.0000000000018933

**Published:** 2020-01-24

**Authors:** Xiaoxiao Lu, Xianming Wu, Xingrong Wu, Panbi Chen, Jin Cui

**Affiliations:** a Acupuncture and Tuina School, Chengdu University of Traditional Chinese Medicine, Chengdu; b Guizhou University of Traditional Chinese Medicine, Guiyang, China.

**Keywords:** acupoint catgut embedding, asthma, protocol, systematic review

## Abstract

**Background:**

Asthma exacerbations can do a lot of harm to the patients and consume large amounts of medical resources. Although some studies have evaluated acupuncture for asthma in children, few studies have estimated acupoint catgut embedding therapy. We aim to assess the efficacy and safety of acupoint catgut embedding for asthma in adults.

**Methods:**

The following databases will be searched from inception to December, 2019: Medline, Cochrane Central Register of Controlled Trials (CENTRAL), PubMed, EMBASE, the China National Knowledge Infrastructure Database (CNKI), the Chinese Scientific Journal Database (VIP database), and the Wan-fang database. We will also search relevant conference abstracts, and registers of clinical trials. Two reviewers will independently undertake study selection, data extraction, and quality assessment. Data will be synthesized by either the fixed-effects or random-effects model according to a heterogeneity test. The Revman V5.3 software will be used to conduct meta-analysis.

**Results:**

The primary outcome was lung function, forced expiratory volume in one second and forced vital capacity (FVC) were the most commonly used parameters to evaluate lung function. The safety assessment includes the incidence of adverse events.

**Conclusion:**

This systematic review will retrieve clinical randomized controlled trials (RCT) on acupoint catgut embedding for asthma in 7 databases, aiming to describe and update existing evidence on the efficacy and safety of acupoint catgut embedding for asthma in adults.

PROSPERO registration number: CRD42019148401.

**Key Points**

- Study selection, data extraction, and quality assessment will be performed independently by 2 researchers, which will ensure that all relevant studies are included without personal biases.
- Medical databases in other languages (eg, Japanese and Korean) will not be searched because of language barriers, so language bias may exist.
- There may be high heterogeneity from the various evaluation standards in different acupuncture therapies.

## 1 Introduction

Asthma is a heterogeneous disease, usually characterized by chronic airway inflammation. It is defined by the history of respiratory symptoms such as wheeze, shortness of breath, chest tightness, and cough that vary over time and in intensity, together with variable expiratory airflow limitation.^[1]^ Airflow limitation may later become persistent. Clinically, asthmatics exhibit recurrent episodes of wheeze, cough, chest tightness, and shortness of breath.^[2]^ Total 20-year (from 2019 to 2038) direct costs associated with uncontrolled asthma are estimated to be $300.6 billion. When indirect costs are added, total economic burden will be $963.5 billion.^[3]^ While asthma incidence and prevalence are higher in children, morbidity, and mortality are higher in adults. Although some studies have evaluated acupuncture for asthma in children, few studies have estimated acupoint catgut embedding therapy in adults.

The global initiative for asthma advice that consider step down once good asthma control has been achieved and maintained for about 3 months, to find the patient's lowest treatment that controls both symptoms and exacerbations. In traditional Chinese medicine, acupoint catgut embedding therapy has been used for the treatment of several diseases such as musculoskeletal pain, obesity, and chronic urticaria. Many case reports have been documented in China, and a well-designed study examining the efficacy and safety of acupoint catgut-embedding therapy is desirable.

## 2 Criteria for including study inclusion

### 2.1 Type of studies

Randomized controlled trials (RCTs) in English or Chinese will be included without restriction of publication type. Quasi-RCTs will be excluded as they are not truly randomized, and there is a greater risk of selection bias in trials in which allocation is not adequately concealed.

### 2.2 Types of participants

Patients with asthma, older than 18 years of age, regardless of sex, race or educational and economic status, according to the following criteria:

(1) Global strategy for asthma management and prevention (2019 update): definition, description, and diagnosis of asthma
(2) The Chinese experts’ consensus on the evaluation and management of asthma

### 2.3 Types of interventions

Interventions in the observation group included simple acupoint catgut embedding and acupoint catgut embedding combined with other therapies. The control intervention group included no active intervention, sham acupoint catgut embedding, and drugs. We will include the following comparisons:

(1) Acupoint catgut embedding is compared with other therapies (acupuncture, electroacupuncture, cupping, auricular point application, etc)
(2) Acupoint catgut embedding combined with other therapies compared with other therapies
(3) Comparison of acupoint catgut embedding and no active intervention
(4) Comparison of acupoint catgut embedding and sham acupoint catgut embedding

If there were multiple intervention groups, and each intervention group will be compared to a single control group.

### 2.4 Types of outcomes

The primary outcome was lung function, forced expiratory volume in one second and forced vital capacity (FVC) were the most commonly used parameters to evaluate lung function. Other outcomes of interest were symptoms (eg, sputum volume), medication usage, and adverse events.

### 2.5 Search methods for identification of studies

Design and implementation of a search strategy will be based on the Cochrane handbook instruction manual.

### 2.6 Electronic search

An electronic search strategy will be designed to search relevant references in the Cochrane Central Register of Controlled Trials, Medline, PubMed, EMBASE, the China National Knowledge Infrastructure Database (CNKI), the Chinese Scientific Journal Database, and the Wan-fang database. The search will be performed in English and Chinese. The following terms will be used: acupoint catgut embedding; catgut embedding; catgut implantation; (Table 1 details of the search strategy for EMBASE). The search terms will be translated into Chinese when reviewers search the Chinese databases. The following literature sources in Chinese will also be searched: dissertations in CNKI, and conference papers in the China Conference Paper Database. Relevant references cited in selected studies will also be searched.

**Table 1:**
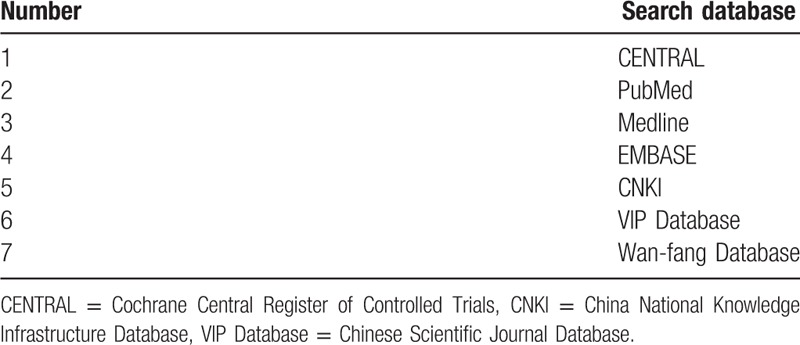
Electronic search.

### 2.7 Searching other resources

The reference lists of studies and systematic reviews related to asthma and acupuncture will be examined for additional trials. Reference texts including integrative/alternative and complementary medicine textbooks and clinical guidelines for relevant trials will also be searched manually.

### 2.8 Data collection and analysis

#### 2.8.1 Selection of studies

**Figure 1:**
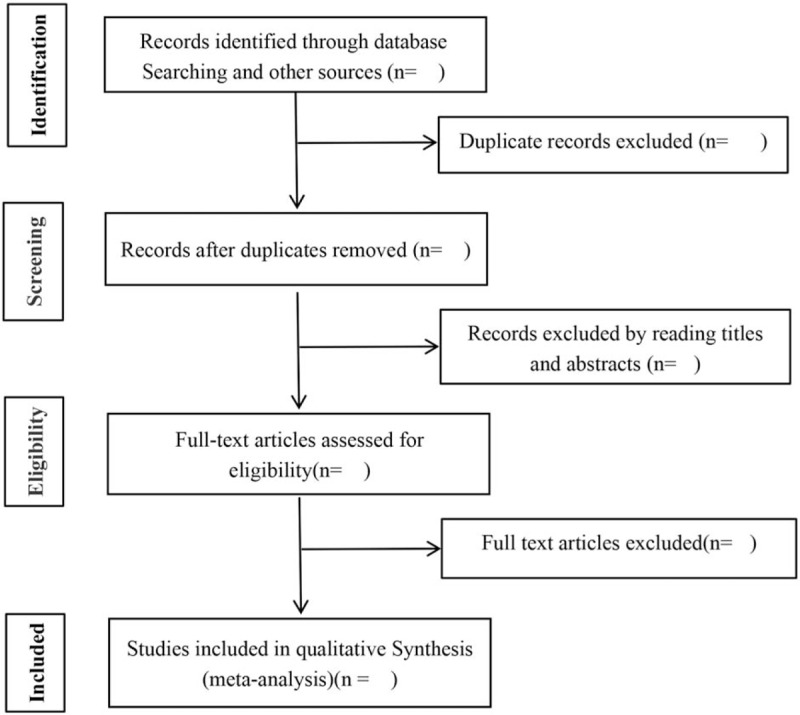
Flow chart of trial selection process for this systematic review.

Our screening process will be discussed and developed before the selection of studies, and the results will be curated using EndNote software. According to the inclusion and exclusion criteria, riate studies will identified and collected by 2 independent reviewers (XL and XMW) after reading the titles, abstracts or full texts. The authenticity of this inclusion analysis will be verified by retrieving published protocols, contacting the first author or correspondent author. Any disagreements over inclusion will be decided by a third reviewer (JC) after discussion. Details of the selection process are shown in Figure 1.

### 2.9 Data extraction and management

All reviewers discussed and produced a trial data extraction form based on the Cochrane Handbook. Two independent reviewers (XL and XMW) extracted data from the selected studies. We will extract the following data:

(1) General information: the first author, time of publication, the source/journal.
(2) Methods: study design, study duration, country, study setting, withdrawals/drop-outs.
(3) Participants: sample size, meanage, agerange, gender, severity of asthma, baseline lung function, inclusion criteria, and exclusion criteria.
(4) Interventions: number and name of acupoints, duration of treatment, frequency of treatment, description of therapists’ qualifications.
(5) Outcomes: primary and secondary outcomes specified and collected, and time points reported.
(6) Notes: funding sources, and notable conflicts of interest of trial authors.

Any disagreement regarding data extraction will be resolved through discussion with the third reviewer (JC). If there is incomplete data, the published protocols will be retrieved, and the first author or corresponding author will be contacted.

### 2.10 Assessment of risk of bias in included studies

The risk of bias in each study will be assessed by 2 independent authors (XL and XMW) using the criteria outlined in the Cochrane Handbook for Systematic Reviews of Interventions. There are 7 domains in the Cochrane “Risk of bias tool”: random sequence generation, allocation concealment, blinding of participants and personnel, blinding of outcome assessment, incomplete outcome data, selective outcome reporting, and other bias. We will grade each potential trial of bias as low, unclear or high, and the judgement with a justification will be filled in the “Risk of bias” table. Any disagreement will be resolved by discussion or by involving an arbiter (JC).

If the number of included studies is more than 10, we will generate funnel plots to detect reporting biases and small-study effects.

### 2.11 Assessment of reporting biases

All reviewers retrieve the published trial protocols, contact the first author or correspondent author of the study for information on selective reports. According to the criteria of the “Critical Risk Assessment Tool” of the Cochrane Handbook (V.5.1.0), the collected results will be judged as having a “high risk bias,” “low risk bias.” and “unclear risk bias.” In the subsequent meta-analysis, if more than 10 trials are included, a funnel plot will be used to evaluate potential publication bias. The asymmetry of the funnel plot may be due to publication or related bias, or it may be due to systematic differences between the various types of research. If the reason for the asymmetry is clear and the clinical diversity of the studies is to be further studied, it should be explained. Because evolution of this graph is subjective, Egger method will also be used to evaluate report bias.

### 2.12 Assessment of heterogeneity

Heterogeneity assessment will be performed before meta-analysis, and the clinical and method heterogeneity will be estimated based on data recorded in the extracted form. Heterogeneity will be calculated using the Mantel–Haenszel *χ*^2^ test. *P*-value <.10 or high *I*^2^ values indicates that the heterogeneity is statistically significant. The Cochrane Handbook divides *I*^2^ values into 4 categories: 0% to 40% represents less or no heterogeneity; 30% to 60% is moderate heterogeneity; 50% to 90% means greater heterogeneity; 75% to 100% corresponds to considerable heterogeneity. The size of the impact, the direction of the outcomes and the strength of the evidence also affect heterogeneity.

### 2.13 Data synthesis

Data synthesis will be performed using Review Manager (V.5.3) statistical software. More than 2 clinical, methodological, and heterogeneous studies will used to perform a meta-analysis to calculate the risk ratio of 95% confidence intervals for the dichotomous data. If the included studies are heterogeneous and have a *P*-value <.10, the risk ratio, weighted mean difference will be calculated using the random-effects model, and conversely calculated by the fixed-effect model.

### 2.14 Subgroup analysis

Subgroup analysis will be based on different types of acupoint catgut embedding therapy, treatment duration, and curative effects. The incidence of adverse events will be statistics and assessed using descriptive methods.

### 2.15 Sensitivity analysis

Sensitivity analysis will be performed if data are sufficient and no errors occur during data input steps. After excluding studies with high risk of bias of blinding and poor methodological quality, meta-analysis will be repeated and the results of the 2 meta-analyses will be compared. Case studies and complete intention-to-treat analysis can be used based on the sample size of the study, the strength of the evidence, and the influence on the pooled effect size.

## 3 Discussion

This review aims to analyze the effects and safety of acupoint catgut embedding for asthma in adults. The systematic review protocol of acupuncture for children with asthma published by Meng Li and colleagues only included articles as of January 1, 2017.^[4]^

When inhaled corticosteroid (ICS) were introduced into asthma management, large improvements were observed in symptom control, lung function and asthma-related mortality decreased. While ICS adherence remains low. ICS augmentation is disproportionate across racial/ethnic groups.^[5]^ ICS treatment duration were significantly associated with adrenal suppression.^[6]^ Therefore, alternative strategies are necessary to treat asthma. This review will provide current evidence on the therapeutic effects of acupoint catgut embedding for asthma, which may benefit practitioners, patients, and policymakers.

## Author contributions

The protocol manuscript was drafted by XL, and revised by JC. The search strategy was formulated by all authors, XL and XMW will independently screen potential studies and extract data from included studies. XRW and Panbi Chen will assess the risk of bias and finish data synthesis. JC will arbitrate any disagreements and ensure that no errors occur during the review. All authors have approved the publication of the protocol.

Xiaoxiao Lu orcid: 0000-0003-2303-7941.
